# 1 K Medicinal Plant Genome Database: an integrated database combining genomes and metabolites of medicinal plants

**DOI:** 10.1093/hr/uhac075

**Published:** 2022-03-23

**Authors:** Xiaojun Su, Lulu Yang, Dongliang Wang, Ziqiang Shu, Yicheng Yang, Shilin Chen, Chi Song

**Affiliations:** 1 Innovative Institute of Chinese Medicine and Pharmacy, Chengdu University of Traditional Chinese Medicine, 611137 Chengdu, Sichuan, China; 2 Wuhan Benagen Technology Company Limited, 430070 Wuhan, Hubei, China; 3 Department of Cell Biology and Genetics, Shenzhen University Health Sciences Center, 1066 Xueyuan Avenue, 518060 Shenzhen, Guangdong, China; 4 China Academy of Chinese Medical Sciences, Institute of Chinese Materia Medica, 100070 Beijing, China

##  

Dear Editor,

Medicinal plants are composed of complex natural compounds with diverse medicinal applications, which makes them crucial resources for drug research. Since the genome of the medicinal plant *Ricinus communis* was first reported in 2010 [1], various medicinal plants have been sequenced and analyzed, including chrysanthemums (*Chrysanthemum nankingense*) [2], Chinese goldthread (*Coptis chinensis*) [3], *Aristolochia fimbriata* [4], and *Taxus* species [5–7]. Many genomic data of medicinal plants are being publicly published [8]. However, there is a lack of a comprehensive database containing the genomes and secondary metabolites of medicinal plants, which will facilitate research on herbal medicine.

We have built the 1 K Medicinal Plant Genome Database (1 K-MPGD, Fig. 1a, http://www.herbgenome.com/) to collect genomic data on herbal plants and information on natural medicinal ingredients in the pharmacopeia. This database provides resources for traditional medical research. It includes BLAST [9], JBrowse [10], primer design, and other data analysis tools. This database is directly managed by Wuhan Benagen Technology Co., Ltd. We will collect, sort out, update, and upload new data every 3 months. A reliable data management system has been developed and all newly released information will be updated on this website. Wuhan Benagen Technology Co., Ltd will continue to update and manage the 1 K-MPGD.

**Figure 1:**
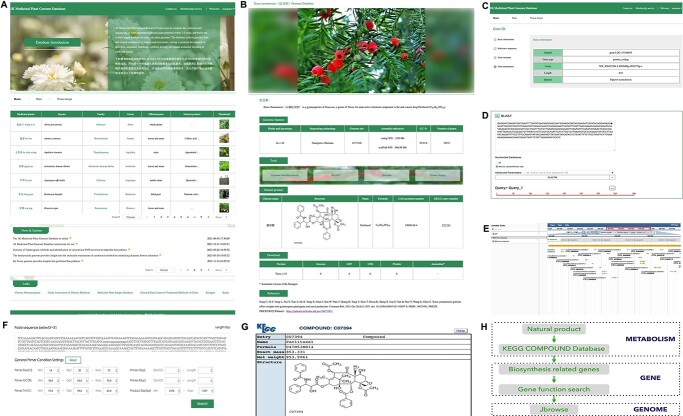
Structure of the 1 K Medicinal Plant Genome Database (1 K-MPGD). **a** Home page of 1 K-MPGD. **b** Page of genomic and metabolic data on medicinal plants. **c** Genome function search tool; **d** BLAST tool. **e** JBrowse tool. **f** Primer design tool. **g** Entry into the KEGG COMPOUND Database through the natural product KEGG entry number. **h** 1 K-MPGD combines metabolism, gene, and genome research.

At present the 1 K-MPGD collects nearly 100 published genomes of medicinal plants, including *Taxus yunnanensis* [6] and other important species (Fig. 1a). Medicinal plant genome data were collected from publicly available genome projects. The data on each species consist of an introduction, genome information, chemical components, downloadable information, and published references (Fig. 1b). For the downloadable data, we have summarized the sequencing platform, genomic size, assembly and annotation results. Genomic data are contained in a FASTA formatted genome file, with a coding sequence (CDS) file available in FASTA format, and a protein data file available in both FASTA and GFF3 formats. The genome function search, BLAST, JBrowse, and primer design tools are linked for further genetic and enzyme-based analyses.

As a central portal for medicinal plant genomics, it provides users with analytic tools such as genome function search (Fig. 1c), BLAST (Fig. 1d), JBrowse (Fig. 1e), and primer design (Fig. 1f), allowing users to search and analyze their target genes. Each natural product of a medicinal plant is linked to the Kyoto Encyclopedia of Genes and Genomes (KEGG) database by KEGG entry number (Fig. 1g). Here, users can search for genes related to biosynthesis of natural products of medicinal plants through the KEGG COMPOUND Database (https://www.kegg.jp/kegg/compound/), and then search for gene sequence, structure, and annotation information by gene name in 1 K-MPGD’s gene function search tool. The JBrowse tool in 1 K-MPGD shows the location of the gene in the species genome. The one-stop integration of metabolism, gene, and genome datasets provides great convenience for medicinal plant research (Fig. 1h).

We plan to collect nearly 1000 genomes [or genome data] of medicinal plants within 5 years. In the future, we will also sequence and assemble some challenging herbal genomes, such as the giant genome of the Liliaceae and polyploid herbal genomes, which will be uploaded to this database. 1 K-MPGD will continue to develop new extension modules to better integrate metabolomic and even transcriptomic data with the genome, and develop more analysis functions embedded in the website. We believe that 1 K-MPGD will become a global and active platform to help researchers and breeders accelerate medicinal plant breeding.

In summary, 1 K-MPGD provides a comprehensive set of omics data and KEGG pathway information for medicinal plants. 1 K-MPGD will be regularly updated with newly published herbal genomes and will be further improved with enhanced functionalities in order to facilitate comparative genomics and synthetic biology research.

## Acknowledgements

This work was supported by the National Key R&D Program of China from the Ministry of Science and Technology of China (grant no. 2021YFE0100900).

## Author contributions

L.Y., S.C., and C.S. designed and managed the project; D.W., Z.S., and Y.Y. constructed the database. X.S. and D.W. collected and analyzed the data. X.S., L.Y., D.W., Z.S., Y.Y., S.C., and C.S. participated in discussions. X.S., L.Y., S.C., and C.S. wrote and revised the manuscript.

## Data Availability

The 1 K-MPGD can be freely accessed at http://www.herbgenome.com/ via the World Wide Web. A reliable data management system has been developed and all newly released information will be updated on this website. Enquiries concerning the database should be directed by email to support@benagen.com.

## Conflict of interest

The authors declare that they have no conflict of interest.
